# Eating behaviors and nutritional habits among young adults using electronic nicotine delivery systems in a multinational cross-sectional study

**DOI:** 10.1186/s12889-025-24318-3

**Published:** 2025-11-03

**Authors:** Saadi M. Saleh, Abdullah Al-Janabi, Moaz Elsayed Abouelmagd, Omar Habib, Siham M. Abdi, Mariam Zainab, Nermin Adly Hassan, DARS Consortium, Alia Alkhayer, Basma Ahmed Mohammed Al-Sammraei, Basma Taher, Cesar Khouday, Labibah L. Alswelhi, Mansour A. Algazar, Mariam Alkeswani, Mohammad Yassin Al Aboud, Nabaa Alturfi, Rawshan Ameen Nassrullah, Sara Najim, Safia M. Abdi

**Affiliations:** 1 Faculty of Medicine, Benha University, Benha, Egypt; 2 Faculty of Medicine, Cairo University, Cairo, Egypt; 3 Faculty of Medicine, Port Said University, Port Said, Egypt; 4 Faculty of Medicine, Ahfad University for Women, Khartoum, Sudan; 5 Faculty of Medicine, Al Faisal University, Riyadh, Saudi Arabia; 6 Department of Forensic Medicine and Clinical Toxicology, Faculty of Medicine of Benha University, Benha, Egypt

**Keywords:** ENDS, Vaping, Eating behavior, MENA, Electronic nicotine delivery systems

## Abstract

**Background:**

Electronic nicotine delivery systems (ENDS) use is rising among young adults in the Middle East and North Africa (MENA) region. While marketed as safer alternatives to smoking, their impact on eating behaviors and nutritional habits remains unclear. This study aims to examine the relationship between ENDS use, tobacco smoking, and eating behaviors among young adults in MENA.

**Methods:**

A cross-sectional study was conducted from December 2023 to February 2024 with participants aged 18–35 from Egypt, Iraq, Jordan, Syria, and Saudi Arabia. Data were collected using an online survey and using the Adult Eating Behavior Questionnaire (AEBQ-Ar). Multiple linear and multinomial logistic regressions were used to assess associations after adjustment for key confounders. All statistical tests were conducted through Jamovi 2.3.2 for Windows 11.

**Results:**

3350 participants answered our questionnaire. ENDS-only users had a higher BMI and reported higher consumption of meals (AOR: 2.08; 95% CI: 1.72, 2.52; *p* < 0.001), spicy food (AOR: 1.82; 95% CI: 1.29, 2.57; *p* < 0.001), coffee (AOR: 5.98; 95% CI: 1.74, 20.56; *p* = 0.005), and fizzy drinks (AOR: 7.23; 95% CI: 2.06, 25.31; *p* = 0.002) compared to non-smokers. ENDS-only users showed significant associations with emotional overeating (β: 0.093; 95% CI: 0.001, 0.186; *p* = 0.048), increased satiety responsiveness (β: 0.179; 95% CI: 0.084, 0.273; *p* < 0.001), and reduced enjoyment of food (β: -0.364; 95% CI: -0.458, -0.269; *p* < 0.001). Tobacco smokers were more likely to consume fatty foods (AOR: 1.61; 95% CI: 1.16, 2.24; *p* = 0.004). Furthermore, tobacco-only smokers, ENDS-only users, and dual-users had higher odds for alcohol consumption compared to non-smokers.

**Conclusion:**

ENDS users exhibit distinct eating behaviors. These behaviors may have long-term implications for their physical health and psychological well-being. Further studies are needed to confirm our findings.

## Introduction

Since its initial introduction in 2003 [1], the use of electronic nicotine delivery systems (ENDS), commonly referred to as e-cigarettes, has grown to reach approximately 4.5% among adult Americans in 2021 [2]. ENDS are devices that heat and vaporize e-liquids that contain nicotine, flavorings, and other compounds to deliver nicotine as claimed in a less dangerous manner than traditional cigarette smoking. While ENDS were initially marketed as a smoking cessation aid, recent research undermines that assertion [3, 4]. The sales and use of ENDS is poorly regulated in most Middle East and North Africa (MENA) countries except for Jordan, Saudi Arabia, and the United Arab Emirates (UAE), while in other countries it’s usually under the same legal framework as tobacco products or even more relaxed regulations due to advertisement of the “benefits” of ENDS [5]. Furthermore, the use of ENDS has increased among youth in the MENA over the years, with rates as high as 10.6% in Egypt [6] and 11.7% in Jordan [7].

The impact of nicotine on body weight has been extensively studied. Nicotine decreases body weight by several mechanisms, including raising metabolic rates, increasing energy expenditure, and suppressing appetite [8]. However, some literature suggests that cigarette smokers eat more fast food and have more cravings for fat and sweets. Furthermore, tobacco smokers consume fewer fruits, vegetables, and healthy meals [9, 10]. Moreover, evidence suggests that body weight tends to increase afr smoking cessation [11].

Recent studies found a higher prevalence of eating disorders (binge eating disorder, and bulimia nervosa), and unhealthy nutritional patterns with higher degrees of nicotine dependency and chronic smoking [9, 12]. However, stressful life events may mediate this relationship; people who experience prolonged stress may be more prone to both cigarette smoking and consuming an unhealthy diet [12].

While studies suggest that some individuals use ENDS to lose weight [13], the existing evidence on the association between ENDS use and weight loss is not yet conclusive [14]. This behavior might be influenced by the common belief that cigarette smoking aids in weight loss. Additionally, recent studies reported conflicting results regarding the association between ENDS use and eating disorders [15, 16]. Few articles have investigated the relationship between ENDS and dietary patterns. Jackson et al. and Ganson et al. found no significant relationship between ENDS use and poor eating behaviors [15, 17]. Ganson et al. found that vaping was associated with higher odds of all eating disorders, like anorexia nervosa and binge eating [15].

To our knowledge, there is a lack of studies examining the relationship between ENDS use and eating behaviors in the world, and especially in the MENA region, especially with the culturally unique eating patterns in the MENA region [18, 19]. In the present study, we aim to investigate the relationship between ENDS use and eating behaviors and nutritional habits among young adults from the MENA region. Furthermore, we expand upon previous research by comparing eating behaviors and nutritional habits among ENDS-only users, tobacco-only smokers, and dual users to non-smokers (neither use ENDS nor smoke tobacco).

## Methods

### Study design and eligibility criteria

We conducted a multinational cross-sectional study in Egypt, Iraq, Jordan, Syria, and the Kingdom of Saudi Arabia (KSA) from December 5th, 2023, to February 25th,2024. Our study included participants aged between 18 and 35 years who currently live in one of the following countries: Egypt, Iraq, Jordan, Syria, and the Kingdom of Saudi Arabia (KSA) with the ability to read and respond to the survey in Arabic. Participants were excluded if they submitted incomplete surveys or did not meet the age or geographic criteria. We chose the 18–35 age group to focus on young adults in the MENA region, as they are key users of ENDS and at a formative stage for eating and lifestyle habits. This group is more behaviorally and socially homogeneous. Participants who did not meet the inclusion criteria were excluded from the study.

### Ethical considerations and approval

Informed consent was obtained from the interested participants at the beginning of the survey, with the possibility of dropping out of the questionnaire at any point without any consequences. The Research Ethics Committee at the Faculty of Medicine, Benha University (REC-FOMBU) in Egypt granted ethical approval with the registration number ‘RC.33.11.2023’.

### Sampling and sample size

We calculated the sample size using Epi Info version 7.2.5.0 on Microsoft Windows 11. Given the lack of similar studies on eating behaviors among ENDS users, we based our effect size on a study by Ganson et al., which reported that 25% of ENDS users were at elevated risk for an eating disorder [15]. With a 5% margin of error and 95% confidence level, the minimum required sample size was 288 participants. To account for the non-random sampling approach, a design effect of 1.5 was applied, increasing the target sample size to 432 participants per country. Ultimately, 3350 participants were included in the final analysis through convenience sampling.

### Data collection

We collected data through an online self-administered Arabic Google Form survey. The survey was distributed by a team of collaborators through various social media platforms by public posts and messaging channels (Facebook, Instagram, Snapchat, Twitter, WhatsApp, Telegram, and email).

Collaborators were recruited from each country to help with data collection. To recruit ENDS users, data collectors posted information about the study in different groups and channels of ENDS users. Interested individuals were contacted to verify if they met the inclusion criteria and, if eligible, were sent the survey link. To prevent duplicate responses, we configured the form to accept only one response per participant.

### Study tools and variables

The questionnaire comprised four primary sections. The first section focused on the sociodemographic and medical history details of the participants. Sociodemographic questions were about age, sex, country of residence, and anthropometric measurements for calculating body mass index (BMI), while medical history questions covered their physical and mental comorbidities, and medications or supplements usage, in addition to questions about lifestyle factors including sleep patterns, and exercise frequency. The second section included questions about the participants’ smoking habits, addressing tobacco smoking and ENDS usage status for both current and former smokers. The third section explores the participants’ nutritional habits by asking about average water consumption per day, average number of meals consumed per day, type of food frequently consumed, and type of drinks frequently consumed. The final section investigates participants’ current eating behavior by completing a 27-item scale of the validated Arabic version of the Adult Eating Behavior Questionnaire (AEBQ-Ar) [17, 18]. The AEBQ-Ar was adapted from the original English version and demonstrated high internal consistency across all subscales (Cronbach’s alpha ranging from 0.70 to 0.85). The AEBQ-Ar retains the same structure as the original version, consisting of 27 items divided into six subscales: hunger, enjoyment of food, emotional overeating, satiety responsiveness, food fussiness, and slowness of eating with minor linguistic adjustments to account for cultural differences Permission to use the AEBQ-Ar in this study was granted by the original authors.*)*

### Data analysis

Data were exported from Google Forms into Microsoft Excel and translated from Arabic to English. The total sample was divided into four groups: (1) non-smokers (neither tobacco smokers nor ENDS users), (2) tobacco-only smokers, (3) ENDS-only users, and (4) dual users. Individuals who reported using any form of combustible tobacco products, including cigarettes, hookah (waterpipe), cigars, and pipes, are classified as tobacco smokers. This category also encompasses users of tobacco-heated products. ENDS-users are defined as participants who reported using any form of ENDS, such as e-cigarettes, mods, pods, e-hookah, e-pipes, or other vaping devices. Non-smokers were defined as participants who reported never using tobacco or ENDS, as well as those who reported former use of either or both products, provided they had stopped at least one month before the survey. ENDS-only users were the participants who currently used ENDS but had never used tobacco or were former tobacco users who had stopped for at least one month. tobacco-only smokers were the participants who currently used combustible tobacco products but had never used ENDS or were former ENDS users who had stopped for at least one month. Dual users: Participants who reported concurrent use of both ENDS and tobacco products at the time of the survey. The data were analyzed using the Jamovi software, version 2.3.2 for Windows. Continuous variables were presented using mean ± standard deviation (SD) for normally distributed variables or median and Interquartile range (IQR) for those non-normally distributed, whereas categorical variables were expressed as frequencies and percentages (%). We used the Shapiro-Wilk test to test data normality, Student’s t-test, Wilcoxon rank sum test, and Kruskal–Wallis one-way analysis of variance to compare continuous variables and chi-squared tests for categorical variables. Multiple linear regression models were used to examine the relationship between product usage status and eating behavior variables, while multinomial and binomial logistic regression were applied to assess the relationship between product usage status and nutritional habits variables. All regression models were adjusted for key confounding factors, including age, sex, country of residence, BMI, exercise frequency, sleep deprivation, number of physical comorbidities, number of psychiatric comorbidities, and medication or supplement use. Analysis was conducted using adjusted odds ratios (AOR), and a confidence interval of 95% (95% CI) was reported. All P values and 95% CI were two-sided and a p-value < 0.05 was considered statistically significant. Corrupt or missing data were identified using Excel functions, and the multiple imputation by chained equations method was performed to account for missing data.

## Results

### Sample characteristics

Among the 3350 participants involved in the final analysis, 58.6% identified as non-smokers, 12.1% were tobacco-only smokers, 22.1% were ENDS-only users, and 7.2% were dual users. The study participants had a median age of 23 (21, 26) years, and an almost balanced male-to-female ratio with females comprising 51% of the sample.

Non-smokers had a lower median age of 23 (21, 26) compared to tobacco-only smokers (24 [22, 27]), and ENDS-only users (24 [21, 26]), (*p* < 0.001). Males constituted the majority of tobacco-only smokers, ENDS-only users, and dual-users (63%, 72%, and 75%, respectively) (*p* < 0.001). Most ENDS-only users were from KSA (58%), followed by Iraq (21%) (*p* < 0.001).

Regarding the BMI, ENDS-only users had the highest BMI among other groups with a median of 25.5 (21.8, 28.4) kg/m2 (*p* < 0.001). Additionally, most of the participants did not engage in physical exercise (49%), and ENDS-only users were the least group to engage in physical exercise compared to other groups (59%) (*p* < 0.001). Dual-users reported more frequent sleep deprivation (56%), followed by tobacco-only smokers (47%), and ENDS-only users (44%), while non-smokers reported the least (43%) (*p* = 0.001).

The presence of physical comorbidities and psychiatric comorbidities differed significantly among groups, with the proportions being 8.6% (*p* < 0.001) for physical comorbidities and 4.9% (*p* = 0.02) for psychiatric comorbidities. Also, the majority of participants did not use any medication/supplements (89.3%). Detailed sociodemographic characteristics are reported in Table 1.

**Table 1:** The study groups’ distribution according to clinical and sociodemographic characteristics

Variable	Non-smoker *N* = 1963 (59%)	Tobacco-only smoke *N* = 405 (12%)	ENDS-only use *N* = 742 (22%)	Dual-use *N* = 240 (7.2%)	*p*-value
Age (years), ***median (q1***,*** q3)***	23 (21, 26)	24 (22, 27)	24 (21, 26)	24 (22, 27)	< 0.001^1^
Sex					< 0.001^2^
Female	1,307 (67%)	150 (37%)	208 (28%)	60 (25%)	
Male	656 (33%)	255 (63%)	534 (72%)	180 (75%)	
Country of residence					< 0.001^2^
Egypt	474 (24%)	26 (6.4%)	31 (4.2%)	21 (8.8%)	
Iraq	603 (31%)	96 (24%)	158 (21%)	99 (41%)	
Jordan	303 (15%)	74 (18%)	55 (7.4%)	32 (13%)	
Saudi Arabia	376 (19%)	17 (4.2%)	429 (58%)	17 (7.1%)	
Syria	207 (11%)	192 (47%)	69 (9.3%)	71 (30%)	
Body mass index (kg/m ^2^)	23.8 (21.2, 26.6)	24.6 (21.5, 28.0)	25.5 (21.8, 28.4)	24.3 (21.5, 27.7)	< 0.001^1^
Frequency of exercise					< 0.001^2^
No exercise	905 (46%)	164 (40%)	436 (59%)	133 (55%)	
1–2 times/week	532 (27%)	106 (26%)	165 (22%)	47 (20%)	
3–5 times/week	446 (23%)	111 (27%)	115 (15%)	52 (22%)	
> 5 times/week	80 (4.1%)	24 (5.9%)	26 (3.5%)	8 (3.3%)	
Sleep deprivation (yes)	841 (43%)	191 (47%)	325 (44%)	134 (56%)	0.001^2^
Number of physical comorbidities					
0	1,773 (90%)	365 (90%)	713 (96%)	211 (88%)	< 0.001^2^
1	165 (8.4%)	34 (8.4%)	27 (3.6%)	23 (9.6%)	
2	25 (1.3%)	6 (1.5%)	2 (0.3%)	6 (2.5%)	
Number of psychiatric comorbidities					0.029^2^
0	1,863 (95%)	379 (94%)	722 (97%)	222 (92%)	
1	80 (4.1%)	22 (5.4%)	17 (2.3%)	14 (5.8%)	
2	20 (1.0%)	4 (1.0%)	3 (0.4%)	4 (1.7%)	
Medications/supplements use (yes)	241 (12%)	49 (12%)	38 (5.1%)	29 (12%)	< 0.001^2^

^1^ Kruskal-Wallis rank sum test

^2^ Pearson’s Chi-squared test

### Eating behavior associated with product use

Compared to non-smokers, after adjusting for age, sex, country of residence, BMI, frequency of exercising, sleep deprivation, number of physical comorbidities, number of psychiatric comorbidities, and medications/supplements use (Table 2), a negative association was found between ENDS-only use and enjoyment of food (β: −0.364; 95% CI: −0.458, −0.269; *p* < 0.001). Similarly, a negative association was found between tobacco-only smokers and enjoyment of food (β: −0.146; 95% CI: −0.259, −0.032; *p* = 0.012).

**Table 2:** Eating behavior among different product users compared to Non-smokers

Eating Behavior Domains	Unadjusted β (95% CI)	*p*-value	Adjusted β (95% CI)	*p*-value
Enjoyment of food				
Tobacco smoke only	0.0541 (−0.0518, 0.16)	0.317	−0.14641(−0.25997, −0.0329)	0.012
ENDS use only	−0.3268 (−0.4105, −0.243)	< 0.001	−0.36418 (−0.45891, −0.2694)	< 0.001
Dual use	0.0579 (−0.0748, 0.191)	0.392	−0.07717 (−0.2133, 0.059)	0.266
Emotional over-eating				
Tobacco smoke only	−0.1192 (−0.225, −0.013)	0.028	−0.1612 (−0.2724, −0.04996)	0.005
ENDS use only	0.2559 (0.172, 0.3398)	< 0.001	0.09363 (0.000827, 0.18644)	0.048
Dual use	−0.0835 (−0.217, 0.0496)	0.219	−0.08769 (−0.2211, 0.04568)	0.197
Food fussiness				
Tobacco smoke only	−0.1312 (−0.238, −0.0248)	0.016	0.00308 (−0.112, 0.11819)	0.958
ENDS use only	0.2324 (0.148, 0.3164)	< 0.001	0.07667 (−0.0194, 0.17271)	0.118
Dual use	0.0105 (−0.123, 0.1438)	0.877	0.06067 (−0.0773, 0.19868)	0.389
Slowness in eating				
Tobacco smoke only	−0.151 (−0.2579, −0.0446)	0.005	−0.05589 (−0.1721, 0.0603)	0.346
ENDS use only	0.137 (0.0526, 0.221)	0.001	0.18368 (0.0867, 0.2806)	< 0.001
Dual use	−0.113 (−0.2465, 0.0207)	0.098	0.00237 (−0.137, 0.1417)	0.973
Hunger				
Tobacco smoke only	0.0364 (−0.0705, 0.143)	0.504	−0.0176 (−0.13471, 0.0995)	0.769
ENDS use only	0.1035 (0.019, 0.188)	0.016	0.011 (−0.08672, 0.1087)	0.825
Dual use	0.1105 (−0.0235, 0.245)	0.106	0.0429 (−0.0975, 0.1834)	0.549
Satiety responsiveness				
Tobacco smoke only	−0.197 (−0.3036, −0.0904)	< 0.001	0.0411 (−0.0722, 0.1543)	0.477
ENDS use only	0.1259 (0.0417, 0.2101)	0.003	0.179 (0.0844, 0.2735)	< 0.001
Dual use	0.0514 (−0.0821, 0.185)	0.45	0.2177 (0.0819, 0.3536)	0.002

Dual users are participants who use both ENDS and tobacco

Adjusted β: Outcomes are based on regression models that adjusted for factors including age, sex, country of residence, BMI, frequency of exercising, sleep deprivation, number of physical comorbidities, number of psychiatric comorbidities, and medications/supplements use

The reference is the group using neither ENDS nor tobacco

*ENDS* electronic nicotine delivery system

Tobacco-only smokers had an inverse association with emotional overeating (β: −0.161; 95% CI: −0.272, −0.049; *p* = 0.005) compared to non-smokers. In contrast, ENDS-only users had a positive relation with emotional overeating (β: 0.093; 95% CI: 0.000827, 0.186; *p* = 0.048). Besides, no significant associations were found between food fussiness and product use (tobacco, ENDS, dual) (*p* > 0.05). ENDS-only users had a significant positive association with slowness in eating compared to non-smokers (β: 0.183; 95% CI: 0.086, 0.2806; *p* < 0.001).

Regarding the hunger domain, no significant associations were found between hunger and product use (tobacco, ENDS, dual) (*p* > 0.05). Compared to non-smokers, ENDS-only users had a significant positive association with satiety responsiveness (β: 0.179; 95% CI: 0.084, 0.273; *p* = 0.003).

### Nutritional habits associated with product use

After adjusting for age, sex, country of residence, BMI, frequency of exercising, sleep deprivation, number of physical comorbidities, number of psychiatric comorbidities, and medications/supplements used (Table 3), we found that ENDS-only users significantly consumed more meals per day compared to non-smokers (adjusted odds ratio (AOR): 2.083, 95% CI: 1.723, 2.519; *p* < 0.001). Additionally, ENDS-only users tended to consume spicy food more frequently than non-smokers (AOR: 1.822; 95% CI: 1.289, 2.574; *p* < 0.001). On the other hand, tobacco-only smokers consume more frequently fatty food (AOR: 1.611; 95% CI: 1.159, 2.237; *p* = 0.004) compared to non-smokers.

**Table 3:** Nutritional habits among different product users compared to Non-smokers

Nutritional Habits Domains	OR (95% CI)	*p*	AOR (95% CI)^*^	*p*
Number of meals consumed/day^a^				
Tobacco smoke only	1.083 (0.882, 1.33)	0.448	0.919 (0.733, 1.151)	0.462
Ends use only	2.387 (2.029, 2.81)	< 0.001	2.083 (1.723, 2.519)	< 0.001
Dual use	0.969 (0.749, 1.25)	0.808	0.849 (0.646, 1.113)	0.237
Average water consumption/day ^a^				
Tobacco smoke only	1.37 (1.13, 1.67)	0.001	1.026 (0.83, 1.269)	0.809
Ends use only	2.19 (1.89, 2.54)	< 0.001	1.669 (1.4, 1.99)	< 0.001
Dual use	1.32 (1.04, 1.68)	0.025	1.028 (0.795, 1.329)	0.834
Frequently consumed type of food ^b^				
Healthy food				
Tobacco smoke only	0.6991 (0.4896, 0.998)	0.049	0.5519 (0.37058, 0.8219)	0.003
Ends use only	0.8147 (0.6177, 1.074)	0.147	0.9014 (0.65831, 1.2342)	0.517
Dual use	0.7934 (0.5222, 1.205)	0.278	0.7184 (0.45742, 1.1284)	0.151
Fatty food				
Tobacco smoke only	1.8446 (1.3864, 2.454)	< 0.001	1.611 (1.15981, 2.2378)	0.004
Ends use only	1.8874 (1.4862, 2.397)	< 0.001	1.0645 (0.79912, 1.4179)	0.669
Dual use	1.6133 (1.1277, 2.308)	0.009	1.0629 (0.71785, 1.5738)	0.761
Spicy food				
Tobacco smoke only	1.106 (0.7108, 1.721)	0.655	1.298 (0.7943, 2.1211)	0.298
Ends use only	2.9183 (2.2024, 3.867)	< 0.001	1.8223 (1.28986, 2.5745)	< 0.001
Dual use	0.9111 (0.5089, 1.631)	0.754	0.9614 (0.51924, 1.7801)	0.9
Desserts				
Tobacco smoke only	1.1433 (0.744, 1.757)	0.541	0.9656 (0.59562, 1.5655)	0.887
Ends use only	0.8549 (0.5771, 1.266)	0.434	0.8666 (0.55468, 1.354)	0.529
Dual use	0.8769 (0.4903, 1.568)	0.658	0.8266 (0.44651, 1.5301)	0.544
Dairy products				
Tobacco smoke only	1.0902 (0.6651, 1.787)	0.732	0.9007 (0.52113, 1.5566)	0.708
Ends use only	1.5438 (1.0708, 2.226)	0.02	0.9468 (0.61528, 1.4569)	0.804
Dual use	0.7423 (0.3661, 1.505)	0.409	0.6624 (0.31635, 1.3871)	0.275
Frequently consumed drinks ^c^				
Frequently consumed type of drinks				
Water				
Tobacco smoke only	0.491 (0.117, 2.061)	0.331	0.72327 (0.15685, 3.33529)	0.678
Ends use only	2.619 (0.661, 10.378)	0.171	1.92709 (0.44491, 8.34707)	0.38
Dual use	0.656 (0.106, 4.077)	0.651	0.93745 (0.13929, 6.3092)	0.947
Coffee				
Tobacco smoke only	2.782 (1.179, 6.562)	0.019	2.53242 (0.98546, 6.50777)	0.054
Ends use only	8.65 (2.685, 27.867)	< 0.001	5.976 (1.73682, 20.56204)	0.005
Dual use	2.629 (0.805, 8.591)	0.11	2.84121 (0.81898, 9.85683)	0.1
Tea				
Tobacco smoke only	1.439 (0.606, 3.417)	0.41	1.34592 (0.51937, 3.48787)	0.541
Ends use only	4.684 (1.45, 15.13)	0.01	3.22023 (0.93278, 11.11713)	0.064
Dual use	2.116 (0.648, 6.912)	0.215	1.76831 (0.50807, 6.15456)	0.37
Herbal				
Tobacco smoke only	7.688 (2.888, 20.469)	< 0.001	2.13842 (0.73301, 6.23844)	0.164
Ends use only	2.742 (0.616, 12.208)	0.186	1.99718 (0.4112, 9.70015)	0.391
Dual use	2.748 (0.617, 12.237)	0.185	0.89663 (0.18584, 4.32606)	0.892
Fizzy drinks				
Tobacco smoke only	1.848 (0.746, 4.581)	0.185	2.0036 (0.73154, 5.4876)	0.176
Ends use only	13.766 (4.228, 44.816)	< 0.001	7.22577 (2.06256, 25.31407)	0.002
Dual use	4.162 (1.246, 13.903)	0.02	3.23118 (0.89602, 11.65214)	0.073
Citrus (fruit) drinks				
Tobacco smoke only	1.474 (0.565, 3.847)	0.428	1.74888 (0.60805, 5.03019)	0.3
Ends use only	2.947 (0.845, 10.276)	0.09	3.35724 (0.89284, 12.62385)	0.073
Dual use	1.265 (0.33, 4.85)	0.731	1.68516 (0.41102, 6.90909)	0.469
Milk				
Tobacco smoke only	0.907 (0.332, 2.479)	0.85	1.24229 (0.41523, 3.71672)	0.698
Ends use only	11.922 (3.626, 39.202)	< 0.001	6.91901 (1.95198, 24.52516)	0.003
Dual use	1.038 (0.266, 4.056)	0.957	1.38131 (0.33112, 5.7623)	0.658
Alcohol consumption ^d^				
Tobacco smoke only	9.0803 (5.52033, 14.9361)	< 0.001	3.673 (2.1453, 6.28715)	< 0.001
Ends use only	3.139 (1.8437, 5.3445)	< 0.001	2.549 (1.4179, 4.58169)	0.002
Dual use	9.4437 (5.41151, 16.4802)	< 0.001	4.877 (2.6788, 8.87798)	< 0.001

Dual users are participants who use both ENDS and tobacco

*OR* Odds ratio, *AOR* Adjusted odds ratio, *ENDS* Electronic nicotine delivery system

^*^AOR: Outcomes are based on regression models that adjusted for factors including age, sex, country of residence, BMI, frequency of exercising, sleep deprivation, number of physical comorbidities, number of psychiatric comorbidities, and medications/supplements use.

^a^Ordinal logistic regression

^b^Multinomial logistic regression with ‘non-specific type of food’ as a reference

^c^Multinomial logistic regression with ‘non-specific type of drinks’ as a reference

^d^Binomial logistic regression with ‘not frequently consume alcohol’ as a reference

ENDS-only users significantly consumed more coffee (AOR: 5.976; 95% CI: 1.736, 20.562; *p* = 0.005), fizzy drinks (AOR: 7.225; 95% CI: 2.062, 25.314; *p* = 0.002), and milk (AOR: 6,919; 95% CI: 1.951, 24.525; *p* = 0.003) in comparison with non-smokers. Furthermore, tobacco-only smokers, ENDS-only users, and dual-users had higher odds for alcohol consumption compared to non-smokers (AOR: 3,673; 95% CI: 2.145, 6.287; *p* < 0.001), (AOR: 2.549; 95% CI: 1.417, 4.581; *p* = 0.002), and (AOR: 4.877; 95% CI: 2.678, 8.877; *p* < 0.001) respectively. ENDS-only users consumed more water daily than non-smokers (AOR: 1.669; 95% CI: 1.400; 1.990; *p* < 0.001).

## Discussion

This study examined MENA young adults’ reported eating behavior and nutritional habits by ENDS use, tobacco smoke, and dual use. While previous work in this area has studied the risk for eating disorders, the current study adds to the literature by studying the relationship between various eating behaviors, nutritional habits, and product use (ENDS-only use, tobacco-only smoke, and dual-use). We found that ENDS-only users and tobacco-only smokers had a negative association with the enjoyment of food, which is consistent with previous studies on how smoking negatively affects taste buds and taste sensation in general [20–25]. They also showed a tendency for emotional overeating more than non-smokers, which is supported by the literature on the positive relationship between ENDS use and eating disorders [15, 26]. It is also worth noting that ENDS-only users had higher satiety responsiveness and slower eating habits. Nicotine is known to suppress hunger and satiety responsiveness, and a percentage of people start smoking to lose weight may start using it in the hope that it helps them lose weight [27–29]. This acute action of ENDS does not contradict the long-term actions of increased eating disorders, emotional eating, or high-energy foods and drinks [15]. The long-term effects of ENDS use are likely dependent on multiple factors like comorbid psychological conditions, environment, and individual variations which may explain conflicting literature on ENDS use and eating habits and outcomes.

Nicotine binds to nicotinic acetylcholine receptors (nAChRs) on pro-opiomelanocortin (POMC) neurons in the hypothalamus, acutely increasing melanocortin signaling and catecholamine release; this combination suppresses appetite and raises energy expenditure [30]. We also found that ENDS-only users had the highest BMI and were less likely to engage in physical exercise compared to other groups. These results resonate with other cross-sectional studies [14]. However, the results of the longitudinal observational study of Alqahtani et al. found e-cigarette use to be associated with lower BMI [31]. These results do not contradict the results of our study, as ENDS use may lower BMI among smokers on the long term but be more prevalent among people with higher BMI [32]. In our cross-sectional sample, ENDS-only users exhibited a higher mean BMI. Because the study lacks pre-ENDS weight data and a longitudinal design, this association could reflect the sample characteristics and self-selection or residual confounding rather than causal effects. We therefore interpret the BMI finding cautiously and highlight the need for longitudinal studies that control for baseline adiposity and lifestyle factors.

In this cross-sectional study, ENDS-only users reported consuming more meals, spicy food, coffee, fizzy drinks, and milk than non-smokers. Although nicotine is typically associated with appetite suppression and weight loss, cross-sectional data, including our own, sometimes show higher BMI among smokers and ENDS users [14, 27]. This apparent contradiction may stem from behavioral or compensatory eating habits that develop over time, offsetting nicotine’s anorexigenic effects and leading to heavy smokers having higher obesity rates than light smokers and non-smokers [11].

Evidence from Wen et al. suggests that spicy food consumption is positively associated with increased cigarette smoking [33]. Furthermore, Ma and Lee found that smokers had a higher salt preference and salt-related eating behaviors than non-smokers which increased with cigarette consumption. One potential explanation for this preference for strong tastes like salty and spicy is a diminished taste perception specifically for salty food, as compared to other types of food [20] and elevated taste threshold due to the damage of tongue papillae associated with ENDS use and tobacco smoking [21, 23, 24, 34].

Tobacco-only smokers tended to consume more fatty food which was reported in previous studies [9, 35]. Chao et al. reported that Current tobacco smokers consumed more high-fat foods (*p* = 0.02) and more fast-food fats (*p* = 0.001) compared to never-smokers [9].

ENDS users in our sample showed higher daily consumption of water compared to non-smokers. This can be explained by xerostomia (dry mouth) caused by ENDS use. A previous meta-analysis reported the prevalence of xerostomia to be 33% among e-cigarette users [36]. Consequently, the increased water consumption observed among ENDS users may be attributed to their need to hydrate their tongues in response to xerostomia symptoms caused by ENDS use.

These results on coffee and fizzy drinks resonated with previous studies. Ketcher et al. also found ENDS users to prefer vaping with caffeinated beverages like coffee and soda [37]. Miyoshi et al. also found that specific high-energy drinks like coffee and alcohol increase smoking cravings but not milk [38] this may be due to the interaction between the three addictive behaviors through modulating dopaminergic functions [39] and enhancing cigarette taste [40]. Fagan et al. provided several hypotheses on this relation, including that youth engaging in risky behaviors often participate in multiple risky behaviors, and that Factors like peer pressure, stress, and depression can mediate both vaping and high-energy drink consumption [41].

### Strengths and limitations

Our study had several noteworthy strengths. Firstly, we had a large and diverse sample that represented populations from five countries in the MENA region, which is an area often overlooked in existing literature. This allowed us to consider the influence of different cultures, smoking habits, and nutritional habits when examining smoking-related behaviors. Secondly, our regression analysis took into account known influential variables from previous studies, minimizing the impact of confounding factors. We also carefully addressed the presence of fake data and appropriately handled missing data to ensure the validity of our findings. Lastly, we employed a validated questionnaire, the AEBQ-Ar, to collect data on eating behaviors, ensuring the reliability and accuracy of our measurements.

Although our cross-sectional study provides valuable insights into eating behavior and nutritional habits among different groups - non-smokers, tobacco smokers, ENDS users, and dual users - it’s important to acknowledge key limitations that might impact our results’ interpretations. Firstly, the study design was cross-sectional, which restricts our ability to determine causal relationships or the temporal sequence of events, and our results shall be interpreted with caution, especially those with marginal significance, as they may be prone to higher type I error due to the exploratory nature of our study. However, observational studies are the only applicable design due to ethical considerations of conducting clinical trials and asking participants to smoke. Although we considered psychiatric comorbidities in our analysis, a more comprehensive investigation into the role of stress and depression in mediating our results is necessary. It is important to mention that both product use and eating behaviors were self-reported, which introduces the possibility of response bias and may affect the accuracy of reported outcomes such as alcohol use. Some of the analyses of subgroups in our study shall be interpreted with caution due to limited sample sizes in some groups, leading to a higher possibility of accidental significant findings. We interpreted these findings with caution in the discussion section, highlighting that limitation. Although we conducted adjusted regression models for important confounders, we did not account for important confounders like socioeconomic status, education level, adherence to a pcefic diet, and stress levels due to limited data availability and encourage future studies to explore them. Additionally, smokers often underreport their food intake, which could have influenced our findings [42]. While our findings offer valuable insight into behavioral patterns among users, the results may not fully reflect the prevalence of nicotine product use in the general population. Finally, our reliance on convenience sampling may limit the generalizability of the findings due to potential selection bias. These methods, while necessary for recruiting a large and diverse population across the MENA region, do not guarantee a fully representative sample. To mitigate this limitation, we ensured anonymity, confidentiality, and voluntary participation, which encouraged broader involvement. The large sample size also helps reduce the impact of potential bias, enhancing the study’s robustness.

Future studies shall include larger sample sizes with stratified sampling methods to allow for good representation of smaller subgroups in our study. Additionally, we could not specifically describe the relationship between eating patterns and ENDS use in each country due to limited sample size, opening the chance for future country-specific studies. Additionally, they should assess important missing confounders in our study, like socioeconomic status, and specifically analyze the effects of medications and different comorbidities on such a relationship. Also, it’s advisable to include 24-hour dietary recalls, food frequency questionnaires, or digital food diaries to provide a more comprehensive understanding of nutritional behavior among nicotine product users. We also acknowledge that the absence of biochemical validation, such as cotinine testing is a limitation of our study Future research should also consider incorporating large longitudinal cohorts and case-control studies using biochemical validation methods for nicotine use to assess a stronger level of correlation between the variables we found and ENDS, while experimental animal studies may explain the underlying mechanisms and mediating effects of product use on these relationships, further improving the generalizability and depth of understanding. Furthermore, it’s recommended to use validated screening tools for psychological factors to better capture their potential mediating effects in future studies.

## Conclusion

Our study findings indicate that ENDS users exhibit distinct eating behaviors and nutritional habits, including higher consumption of multiple meals, spicy food, and fatty food, even after accounting for relevant demographic and clinical factors. Additionally, ENDS users have higher odds of consuming high-energy drinks such as coffee and fizzy drinks, as well as alcohol. However, they report lower enjoyment of food and show a tendency for psychological conditions like emotional eating. These observed food habits may have implications for their long-term physical health, weight, and psychological well-being. Our study shed light on the relationship between ENDS use, eating behaviors, and nutritional habits in the MENA region. To establish causal relationships or identify potential mediating factors such as stress, further longitudinal studies are needed.

## Data Availability

The data supporting this study’s findings are available from the corresponding author upon reasonable request.

## Abbreviations

ENDS: Electronic Nicotine Delivery Systems
MENA: Middle East and North Africa
BMI: Body Mass Index
AEBQ-Ar: Arabic Version of the Adult Eating Behavior Questionnaire
AOR: Adjusted Odds Ratio
CI: Confidence Interval
IQR: Interquartile Range
KSA: Kingdom of Saudi Arabia
SD: Standard Deviation
Epi Info: Epidemiological Information Software
ANOVA: Analysis of Variance
